# Estimation of ryegrass (*Lolium*) dry matter yield using genomic prediction considering genotype by environment interaction across south-eastern Australia

**DOI:** 10.3389/fpls.2025.1579376

**Published:** 2025-06-09

**Authors:** Jiashuai Zhu, Khageswor Giri, Zibei Lin, Noel O. Cogan, Joe L. Jacobs, Kevin F. Smith

**Affiliations:** ^1^ Faculty of Science, The University of Melbourne, Parkville, VIC, Australia; ^2^ Agriculture Victoria, AgriBio Centre, Bundoora, VIC, Australia; ^3^ School of Applied Systems Biology, La Trobe University, Bundoora, VIC, Australia; ^4^ Agriculture Victoria, Ellinbank, VIC, Australia; ^5^ Agriculture Victoria, Hamilton, Ellinbank, VIC, Australia

**Keywords:** regional evaluation system, environmental adaptability, sustainable forage production, multi-harvest multi-site trials, genomic selection

## Abstract

Genomic Prediction (GP) considering Genotype by Environment (G×E) interactions was, for the first time, used to assess the environment-specific seasonal performance and genetic potential of perennial ryegrass (*Lolium perenne* L.) in a regional evaluation system across southeastern Australia. The study analysed the Dry Matter Yield (DMY) of 72 base cultivars and endophyte symbiotic effects using multi-harvest, multi-site trial data, and genomic data in a best linear unbiased prediction framework. Spatial analysis corrected for field heterogeneities, while Leave-One-Out Cross Validation assessed predictive ability. Results identified two distinct mega-environments: mainland Australia (AUM) and Tasmania (TAS), with cultivars showing environment-specific adaptation (Base and Bealey in AUM; Platinum and Avalon in TAS) or broad adaptability (Shogun). The G×E-enhanced GP model demonstrated an overall 24.9% improved predictive accuracy (Lin’s Concordance Correlation Coefficient, CCC: 0.542) over the Australian industry-standard best linear unbiased estimation model (CCC: 0.434), with genomic information contributing a 12.7% improvement (CCC: from 0.434 to 0.489) and G×E modelling providing an additional 10.8% increase (CCC: from 0.489 to 0.542). Narrow-sense heritability increased from 0.31 to 0.39 with G×E inclusion, while broad-sense heritability remained high in both mega-environments (AUM: 0.73, TAS: 0.74). These findings support informed cultivar selection for the Australian dairy industry and enable genomics-based parental selection in future breeding programs.

## Introduction

Perennial ryegrass (*Lolium perenne* L.) is a mainstay forage species in temperate agriculture, underpinning the global dairy and livestock sectors. Its widespread adoption stems from desirable characteristics including high digestibility, good grazing tolerance, and adaptability to diverse climatic conditions (Gilliland and Hennessy, 2021; Hannaway et al., 1999; Leddin et al., 2020).

Regional evaluation systems have evolved to assess perennial ryegrass performance within specific agricultural contexts. The Australian Forage Value Index (AU-FVI) was developed to assist farmers in selecting economically suitable cultivars by evaluating their performance across five seasonal periods (Summer, Autumn, Winter, Early Spring and Late Spring) (Leddin et al., 2018). Similar systems have also been developed in New Zealand (NZ-FVI) (Chapman et al., 2017) and Ireland (PPI) (McEvoy et al., 2011). They all underscore the necessity of accurately evaluating relative differences in Dry Matter Yield (DMY) among cultivars.

However, perennial ryegrass DMY exhibits complex temporal and spatial variation patterns, challenging its evaluation. Measurements showed substantial seasonal fluctuations from 1396 DM kg/ha/season during Winter periods to 2183 DM kg/ha/season in Late Spring (Giri et al., 2019) and by both management practices and environmental conditions (Colas et al., 2022). Unlike other dairy systems (Chapman et al., 2017; McEvoy et al., 2011), Australia has experienced an extended period without centralized pasture cultivar evaluations, creating unique challenges when selecting suitable cultivars for a given locality (Leddin et al., 2018). These challenges have prompted the exploration of Genomic Prediction (GP) as a promising solution.

Originally developed for animal breeding by Meuwissen et al. (2001), GP has since found successful applications in plant evaluation, by leveraging genome-wide markers to capture the overall additive genetic variance of traits. Various GP methodologies have been studied, primarily Best Linear Unbiased Prediction (BLUP) and Bayesian frameworks (Arojju et al., 2020a, 2018; Byrne et al., 2017; Cericola et al., 2018; Endelman, 2011; Esfandyari et al., 2020; Faville et al., 2021, 2018, 2016; Fè et al., 2016, 2015; Grinberg et al., 2016; Jahufer et al., 2021; Keep et al., 2020; Konkolewska et al., 2023; Malmberg et al., 2023; Meuwissen et al., 2016). The use of BLUP models that integrate genomic information (GBLUP) has been studied as one of the most promising methodologies for quantitative trait evaluation (Arojju et al., 2018; Cericola et al., 2018; Esfandyari et al., 2020; Faville et al., 2018; Konkolewska et al., 2023).

The potential of GP has been demonstrated in perennial ryegrass. For instance, predictive accuracies for traits with high heritability and low genetic complexity, such as heading date range from 0.75 to 0.90 (Fè et al., 2015; Malmberg et al., 2023). Simulation studies further suggest that GP could accelerate genetic gain by two to three times compared to conventional phenotype-only approaches. This is achieved by reducing breeding cycle time while maintaining accuracy with sufficient marker densities (Arojju et al., 2020b; Barre et al., 2022; Guo et al., 2018; Lin et al., 2016). These findings comprehendingly underscore the potential of GP to enhance evaluation efficiency for perennial ryegrass DMY.

However, genomic relationships and GP have not been fully utilised to better estimate DMY in perennial ryegrass regional evaluation systems, particularly using Multi-Harvest, Multi-Site (MHMS) field trials. This is mainly due to a highly complex genetic nature and limited predictive ability further confounded with Genotype by Environment (G×E) interactions (Arojju et al., 2020b; Bornhofen et al., 2022; Faville et al., 2016; Jahufer et al., 2021; Pembleton et al., 2018). Notably, most phenotypic data relates to the contemporary evaluation of populations in one environment, and even when a historical performance database is used, this phenotypic data typically comes from limited environments (Arojju et al., 2020b; Bornhofen et al., 2022; Faville et al., 2016; Grinberg et al., 2016; Jahufer et al., 2021; Pembleton et al., 2018). This narrow focus restricts the ability to accurately evaluate DMY performance across diverse environmental conditions and compromises the prediction of future progeny performance under novel environments. Moreover, environmental variability often dominates phenotypic responses, as evidenced by shifts in cultivar rankings of DMY performance across environments in Ireland (Conaghan et al., 2008), New Zealand (Chapman et al., 2017), and Australia (Zhu et al., 2023).

Given these complexities, extensive MHMS trial data combined with sophisticated statistical methods that can account for G×E interactions have become essential for accurately assessing DMY performance and predicting cultivar adaptation to specific environments (Chapman et al., 2017; Giri et al., 2019; Kemp, 2011; Leddin et al., 2022, 2018; Zhu et al., 2023). Multiple statistical approaches, such as additive main effects and multiplicative interaction models (Annicchiarico, 1997; Li et al., 2023; Sa’diyah and Hadi, 2016; Smith et al., 2001; Yue et al., 2022) and reaction norm models (Bornhofen et al., 2022), have been developed. Additionally, linear mixed models combined with a Factor Analytic (FA) strategy have emerged as a powerful approach for analysing large-scale MHMS trials and accounting for heterogeneous genetic variances across environments (Burgueño et al., 2008; Piepho, 1998; Smith et al., 2015; Zhu et al., 2023).

Furthermore, unclear breeding histories of commercial cultivars have hampered the usage of pedigree relationships and genomic information in DMY prediction in regional evaluation systems. Perennial ryegrass is a self-incompatible species and breeding practices involving multiple cycles of synthetic population breeding (Pembleton et al., 2016; Wang et al., 2014), creating high heterozygosity and intricate genetic structures. This complexity not only makes it difficult to account for genetic relationships among populations but also leads to predictive performances varying in different breeding programs (Alemu et al., 2024; Arojju et al., 2018; Daetwyler et al., 2012).

To address these challenges, this study presents a comprehensive evaluation of multiple base cultivars across diverse breeding programs and assesses their DMY performance across multiple Australian pasture environments. Each base cultivar, genetically distinct from the others, represents a unique genotype. The efforts collectively deliver reliable performance evaluations that account for G×E interactions, while demonstrating the potential of GP to infer breeding values (as genomic estimated breeding values, GEBVs) and genetic adaptability (as environment-driven genetic responses).

## Materials and methods

### Field trial data

The experimental dataset encompassed 23 MHMS pasture trials conducted between 2008 and 2023 (**Table 1**), managed by the Pasture Trial Network and Australian seed companies, comprising 2,260 plots for 143 cultivars, yielding 47,325 observations across 485 harvest events. Each cultivar is a unique combination of one of 118 genetically distinct base cultivars and one of 13 endophytes. The trials employed row-column designs with block replication, where each cultivar was replicated at least four times, with the Victorian WT (control cultivar) replicated up to eight times in certain trials, following established protocols (Leddin et al., 2018). The harvests spanned five forage seasons: Winter (June and July), Early Spring (August and September), Late Spring (October and November), Summer (December, January, and February), and Autumn (March, April, and May); sites spanned five major Australian dairy economic regions: South West Victoria, Gippsland, Tasmania, Northern Victoria and Southern Riverina, and South Australia.

**Table 1:** The 23 multi-harvest, multi-site trials (perennial ryegrass) from 2008 to 2023, including the economic region of the trial, number of harvests, number of columns and rows (Col_Row), number of base cultivars (Cultivar), number of endophytes (Endo), number of observed seasons (i.e., Winter, Early Spring, Late Spring, Summer, and Autumn), and number of observations (Obs) of each trial.

	Trial	Region	Harvest	Col_Row	Cultivar_Endo	Winter	Early Spring	Late Spring	Summer	Autumn	Obs
1	Ballarat2013	South West Victoria	17	20×8	32 + 7	2	3	3	3	2	2720
2	Ballarat2017	South West Victoria	22	14×8	24 + 10	2	2	3	3	2	2464
3	Ballarat2019	South West Victoria	23	8×7	13 + 5	2	2	3	4	3	1288
4	Casterton2012	South West Victoria	16	30×4	30 + 6	3	3	3	1	1	1920
5	CressyTAS2012	Tasmania	16	30×4	30 + 6	3	2	3	2	3	1920
6	CressyTAS2014	Tasmania	20	12×8	22 + 6	3	2	3	3	3	1920
7	EllinbankVIC2015	Gippsland	22	32×4	28 + 9	3	3	3	3	3	2816
8	ElliottTAS2015	Tasmania	26	12×10	27 + 9	2	2	3	4	3	3120
9	GlenThompson2020	South West Victoria	15	19×4	19 + 9	2	3	3	2	1	1140
10	Hamilton2018	South West Victoria	15	25×4	23 + 9	2	2	3	3	1	1500
11	Howlong2010	Northern Victoria and Southern Riverina	24	14×6	21 + 6	3	3	3	3	3	2016
12	Howlong2011	Northern Victoria and Southern Riverina	24	16×6	24 + 5	4	3	3	3	2	2304
13	Howlong2012	Northern Victoria and Southern Riverina	27	10×6	14 + 5	3	3	3	3	2	1620
14	Howlong2014	Northern Victoria and Southern Riverina	27	14×6	20 + 6	1	3	3	4	3	2268
15	LeongathaVIC2016	Gippsland	15	9×8	18 + 6	2	2	3	2	1	1080
16	Macarthur2019	South West Victoria	20	19×4	16 + 9	2	3	3	4	2	1406
17	MtGambier2016	South Australia	24	42×2	21 + 7	2	2	2	4	3	2016
18	Shepparton2008	Northern Victoria and Southern Riverina	18	9×8	14 + 6	2	3	3	3	2	1296
19	SmithtonTAS2017	Tasmania	20	24×4	24 + 8	1	2	3	4	2	1920
20	Terang2018	South West Victoria	24	14×8	26 + 10	2	3	3	4	2	2688
21	TimboonVIC2015	South West Victoria	20	32×4	28 + 9	3	3	3	4	2	2560
22	TongalaVIC2015	Northern Victoria and Southern Riverina	28	16×8	29 + 9	1	3	3	4	3	3584
23	Warrnambool2020	South West Victoria	22	20×4	20 + 9	3	3	3	4	2	1760

### Spatial analysis and phenotyping

Six frameworks of spatial models: Base, Spatial Fixed, Spatial Fixed Linear, Spatial Random, Spatial Mixed, and Spatial Mixed Linear were tested for their effectiveness in accounting for the spatial variation per trial, considering their successful applications in previous spatial analyses of agricultural field trials (Federer et al., 1997; Gilmour et al., 1997; Hawinkel et al., 2022; Hoefler et al., 2020; Piepho et al., 2008; Smith et al., 2005). These models were fitted using ASReml-R (v3.00) (Butler, 2009; Butler et al., 2009) and assessed using log-likelihood (logLik), Akaike Information Criterion (AIC), Bayesian Information Criterion (BIC), and Mean Absolute Error (MAE). Phenotypes were corrected as response values (***y***) by subtracting the estimated spatial effects via the most optimised Spatial Mixed framework, where the spatial effects were fitted as both fixed and random effects. For further details about the spatial models and their performances, please refer to **Supplementary Material S1** and **Supplementary Table S2**.

### Pool sequencing and population genotyping

The study evaluated 72 ryegrass genotypes, sourced from Australian collections and commercial suppliers, with full germplasm details documented by Zhu et al. (2025). Each genotype represents a genetically distinct ryegrass population. Due to restrictions, the remaining 46 pre-commercial breeding lines at trial sites were not sequenced or genotyped.

Deoxyribonucleic acid (DNA) sequencing utilised a target capture approach with probes designed from SNPs (Single Nucleotide Polymorphisms) mapped to the Kyuss reference genome (Frei et al., 2021). All laboratory procedures and bioinformatics analyses followed previously validated protocols (Zhu et al., 2025), encompassing DNA extraction, library preparation, pool sequencing, and population genotyping derivation from allele frequencies. Key marker quality controls included filtering loci with minor allele frequency (MAF > 5%), ensuring sufficient read depth (RD > 5), limiting sample missing data for (< 20%), and excluding loci with low mapping quality (MQ < 50) or low calling quality (QUAL < 20), resulting 85,903 high-quality SNP markers for further analysis (Zhu et al., 2024).

### Genomic relationships

A Genomic Relationship Matrix (GRM) was constructed following Yang et al. (2010) and adapted for allele frequency format. For a pair of genotypes *j* and *k*, their genomic relationship was calculated as:


(1) $$G_{jk}=\frac{1}{N}\sum_{i}A_{ijk}=\{\begin{array}{c} \frac{1}{N}\sum_{i}\frac{2(x_{ij}-p_{i})(x_{ik}-p_{i})}{\frac{1}{K}\sum_{j}{\left(x_{ij}-p_{i}\right)}^{2}},\;j\neq k\\ 1+\frac{1}{N}\sum_{i}\frac{2x_{ij}^{2}-(1+2p_{i})x_{ij}+p_{i}^{2}}{\frac{1}{K}\sum_{j}{\left(x_{ij}-p_{i}\right)}^{2}},\;j=k \end{array}$$

where *N* is the number of SNPs, *N* is the number of genotypes, $x_{ij}$ is the reference allele frequency for the *i*-th SNP of the *j*-th genotype, and $p_{i}$ is the average reference allele frequency at the *i*-th SNP.

The full rank of the initial GRM was verified via eigenvalue decomposition. The nearest positive definite matrix was obtained using the `nearPD` algorithm in Matrix (v1.7) (Bates et al., 2024) in R (R Core Team, 2025) and inverted for subsequent mixed model analyses. The inverse GRM was formatted as a sparse lower triangular matrix to optimize computational efficiency in ASReml-R (v3.00) (Butler, 2009; Butler et al., 2009).

### Prediction and estimation modelling for dry matter yield

Sets of GP models were fitted using ASReml-R (v3.00) (Butler, 2009; Butler et al., 2009) as Equation 2 to predict the DMY of the 72 ryegrass genotypes. Endophytes (*endo*) were fitted as a fixed component to separate their symbiotic effects from DMY responses (Zhu et al., 2025).


(2) $$y=X_{GP}\beta_{GP}+Zg+\epsilon$$

Where, ***y*** is the vector of spatially corrected phenotypes; ***ϵ*** is the vector of residual errors, ϵ∼N(0,Var(ϵ)) .


$$X_{GP}\beta_{GP}=(\mu ,Tri,Har|Tri,Endo){\left(1,\beta_{Tri},\beta_{Har|Tri},\beta_{endo}\right)}^{'}$$

Where, μ is the *Intercept;* Tri , Har|Tri , Endo are design matrices for the fixed effects of *Trial* ( $\beta_{Tri}$ ), *Harvest* effects within *Trial* ( $\beta_{Har|Tri}$ ), and endophyte effects ( $\beta_{endo}$ ), respectively.

The additive genetic effects (***a***) across harvests and trials were assumed to follow a gaussian distribution of N(0,G) , where ***G*** is the GRM calculated as described above. The overall genetic effects (***g***) and its genetic variances across environments were assumed as ***g*** ~ ***N*** (0, ***K***):


$$K_{0}=[\begin{array}{cc} diag({\text{σ}}_{Tri}^{2}{\left(\Sigma_{Har}^{\text{COR}({\text{λ}}_{Har})}⊗I_{geno}\right)}_{Tri}) & 0\\ 0 & G \end{array}]$$

$$K_{1}=\Gamma_{Tri}\Gamma_{Tri}^{'}⊗{\left(\;\Sigma_{Har}^{\text{COR}({\text{λ}}_{Har})}⊗G\right)}_{Tri}+\Psi_{Tri}⊗{\left(\;\Sigma_{Har}^{\text{COR}({\text{λ}}_{Har})}⊗G\right)}_{Tri},$$

$K=K_{0}$ for models without considering G×E, $K=K_{1}$ for models considering G×E.

Wherein, diag() denotes the diagonal matrix where all off-diagonal values are 0; ${\text{σ}}_{Tri}^{2}$ is the unique variance for each *Trial* (*Tri*); $\sum_{Har}^{\text{COR}({\text{λ}}_{Har})}$ is the order- ${\text{λ}}_{Har}$ autoregressive or ante-dependence variance-covariance matrices for *Harvest* ( Har ), ${\text{λ}}_{Har}\in \{1,2,3\}$ ; $I_{geno}$ denotes the independent and identical genotypic variances; $\Gamma_{Tri}$ denotes the FA loading matrices including order-one (FA1), order-two (FA2), and order-three (FA3) structures, $\Gamma_{Tri}^{'}$ denotes the transpose of $\Gamma_{Tri}$ , and $\Psi_{Tri}$ denotes the unique variance matrix of *Tri* in the FA models; its covariance matrix $\Lambda =\Gamma_{Tri}\Gamma_{Tri}^{'}+\Psi_{Tri}$ ; ⊗ denotes the Kronecker product.

A Best Linear Unbiased Estimation (BLUE) model, which is the current industry standard when evaluating DMY performance for Australian dairy regions (DairyAustralia, 2024), was given as


(3) $$y=X_{BLUE}\beta_{BLUE}+\epsilon$$

Where, ***y*** is the vector of spatially corrected phenotypes; ***ϵ*** is the vector of residual errors, ϵ∼N(0,Var(ϵ)) ; and


$$X_{BLUE}\beta_{BLUE}=(\mu ,Tri,Geno|Tri,Har|Tri,Endo){\left(1,\beta_{Tri},\beta_{geno|Tri},\beta_{Har|Tri},\beta_{endo}\right)}^{T}$$

Where, μ is the *Intercept;* Tri , Geno|Tri, Har|Tri , Endo are design matrices for the fixed effects of *Trial* ( $\beta_{Tri}$ ), genotype ( geno ) effects within *Trial* ( $\beta_{geno|Tri}$ ), *Harvest* effects within *Trial* ( $\beta_{Har|Tri}$ ), and endophyte effects ( $\beta_{endo}$ ), respectively.

All the models using Equations 2 and 3 were assessed based on logLik, AIC, BIC, and Mean Squared Error (MSE) to identify the most appropriate model to account for the genetic variance components.

The prediction was achieved by `predict()` in ASReml-R (v3.00) (Butler, 2009; Butler et al., 2009) and visualized in a biplot using an R package ggplot2 (v3.5.1) (Wickham, 2016). The predictions using $K_{0}$ (GBLUP) and $K_{1}$ (G×EBLUP) for the five ryegrass seasons: Winter, Early Spring, Late Spring, Summer, and Autumn, were obtained following a weighting system by Zhu et al. (2023). The mega-environments, AUM and TAS, were identified by the clustering patterns. Specifically, within each mega-environment, harvests were weighted such that their total weight within a given season summed to 1/5 , ensuring equal seasonal contributions. In G×EBLUP, the mega-environment TAS was identified to include CressyTAS2012, CressyTAS2014, ElliottTAS2015, and SmithtonTAS2017, while the remaining trials were classified as AUM.

The Least Significant Difference (LSD) was calculated to evaluate performance variation within each mega-environment. Within-season LSDs were used to determine significant differences among the base cultivars per season and across-season LSDs were used to determine significant differences across seasons. All these LSDs are calculated at a 5% significance level.

The seasonal estimation was achieved by calculating the linear combination of $\hat{\beta_{BLUE}}$ corresponding to the design matrix $X_{BLUE}$ and averaging over the harvests in the corresponding season.

The goodness-of-fit of the full prediction model (GBLUP and G×EBLUP) and estimation model (BLUE) were assessed by Coefficient of Determination (CoD) and root mean square error (RMSE).

### Genomic estimated breeding values and heritability

Genomic Estimated Breeding Values (GEBVs) were predicted as $\tilde{a}$ through the genomic relationship matrix *G* for both GP models (Equation 2). For the model assuming independent genetic variance structures across environments (using $K_{0}$ ), the narrow-sense heritability ( $h^{2}$ ) was calculated as: $h_{0}^{2}={\text{σ}}_{a}^{2}/({\text{σ}}_{a}^{2}+{\text{σ}}_{\epsilon }^{2})$ , where ${\text{σ}}_{a}^{2}$ is the additive genetic variances captured by *G*, and ${\text{σ}}_{\epsilon }^{2}$ is the residual variances. For the model considering G×E interaction (using $K_{1}$ ), the narrow-sense heritability was given as $h_{1}^{2}={\text{σ}}_{a}^{2}/({\text{σ}}_{a}^{2}+{\text{σ}}_{\lambda }^{2}+{\text{σ}}_{\epsilon }^{2})$ where ${\text{σ}}_{\lambda }^{2}$ is the non-additive G×E genetic variances captured by Λ (in $K_{1}$ of Equation 2).

For models considering G×E interaction, the environment-driven genetic responses (EnvY) for each mega-environment was calculated as: ${\text{EnvY}}_{me}\;=\;\tilde{g}w_{me}^{'}$ , where $\tilde{g}$ represents the overall genetic effects and $w_{me}$ is the weighting vector for *Trial*×*Harvest* combinations in each mega-environment. The broad-sense heritability ( $H^{2}$ ) incorporating the genetic variances of the environments was given as $H^{2}=({\text{σ}}_{a}^{2}+{\text{σ}}_{\lambda E}^{2})/({\text{σ}}_{a}^{2}+{\text{σ}}_{\lambda }^{2}+{\text{σ}}_{\epsilon }^{2})$ , where ${\text{σ}}_{\lambda E}^{2}$ denotes the non-additive genetic variances for specific mega-environment, which is a subset of the total non-additive genetic variances ${\text{σ}}_{\lambda }^{2}$ .

### Cross validation and model performance assessment

Leave-One-Out Cross Validation (LOOCV) was performed to assess prediction accuracy and precision for GEBV and environment-driven genetic responses for GP models (Eq. 2). In each validation round, one genotype was excluded from the training population, the EnvYs were predicted using the reduced dataset, and the prediction accuracy was assessed by comparing the EnvYs against the phenotypes corrected for spatial, endophyte and field effects in the five seasons. For BLUE models (Equation 3), which lack genomic relationships, the phenotypic estimates were compared directly with the spatially corrected phenotypes to assess estimation accuracy and precision. The prediction or estimation precision within each mega-environment or dairy region was assessed using Pearson’s Correlation Coefficient (PCC) and overall prediction accuracy and precision was assessed by Lin’s Concordance Correlation Coefficient (CCC).

## Results

### Genomic relationships

**Figure 1:**
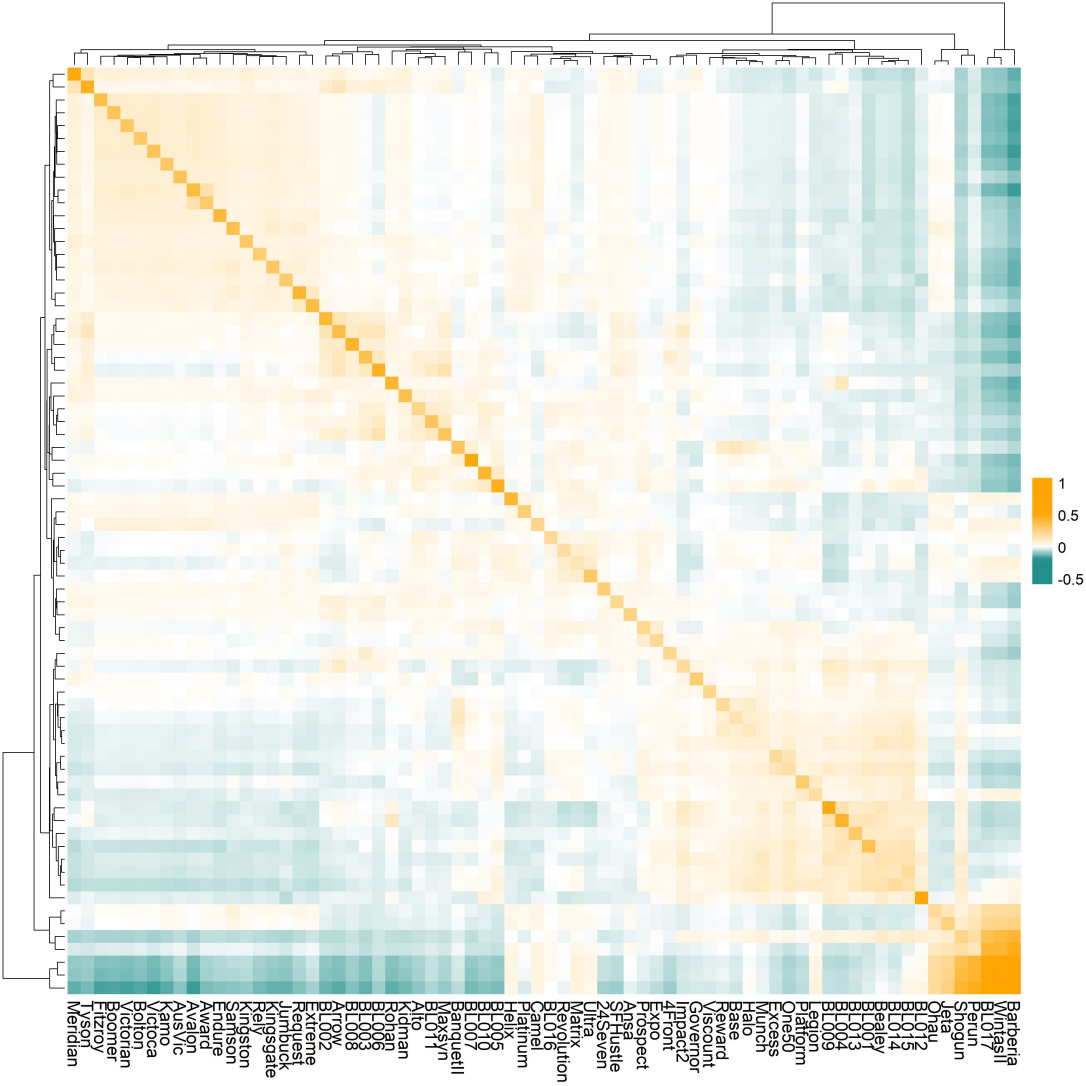
The genomic relationship matrix (GRM) of the 72 ryegrass germplasms with distinct genetic backgrounds. The diagonal values are genomic variances for each germplasm; off-diagonal elements are the genetic covariances between pairs of germplasms. Dendrograms show hierarchical clustering of germplasms based on their genetic relationships.

Genomic relationship analysis characterized the genomic composition of 72 ryegrass accessions (**Figure 1**). The diagonal elements of the GRM ranged from 0.112 (Halo) to 1.372 (Barberia), representing genetic variances for each germplasm. Among these, 71 accessions showed diagonal values less than 1, with only Barberia exceeding 1. The off-diagonal elements ranged from -0.284 to 0.981, representing genetic covariances between pairs of accessions. Hierarchical clustering identified three main clusters corresponding to Italian ryegrass (Barberia to BL017), Boucheanum ryegrass (Perun to Ohau), and perennial ryegrass (BL012 to Meridian), consistent with previous findings (Zhu et al., 2025).

### Model performance

**Figure 2:**
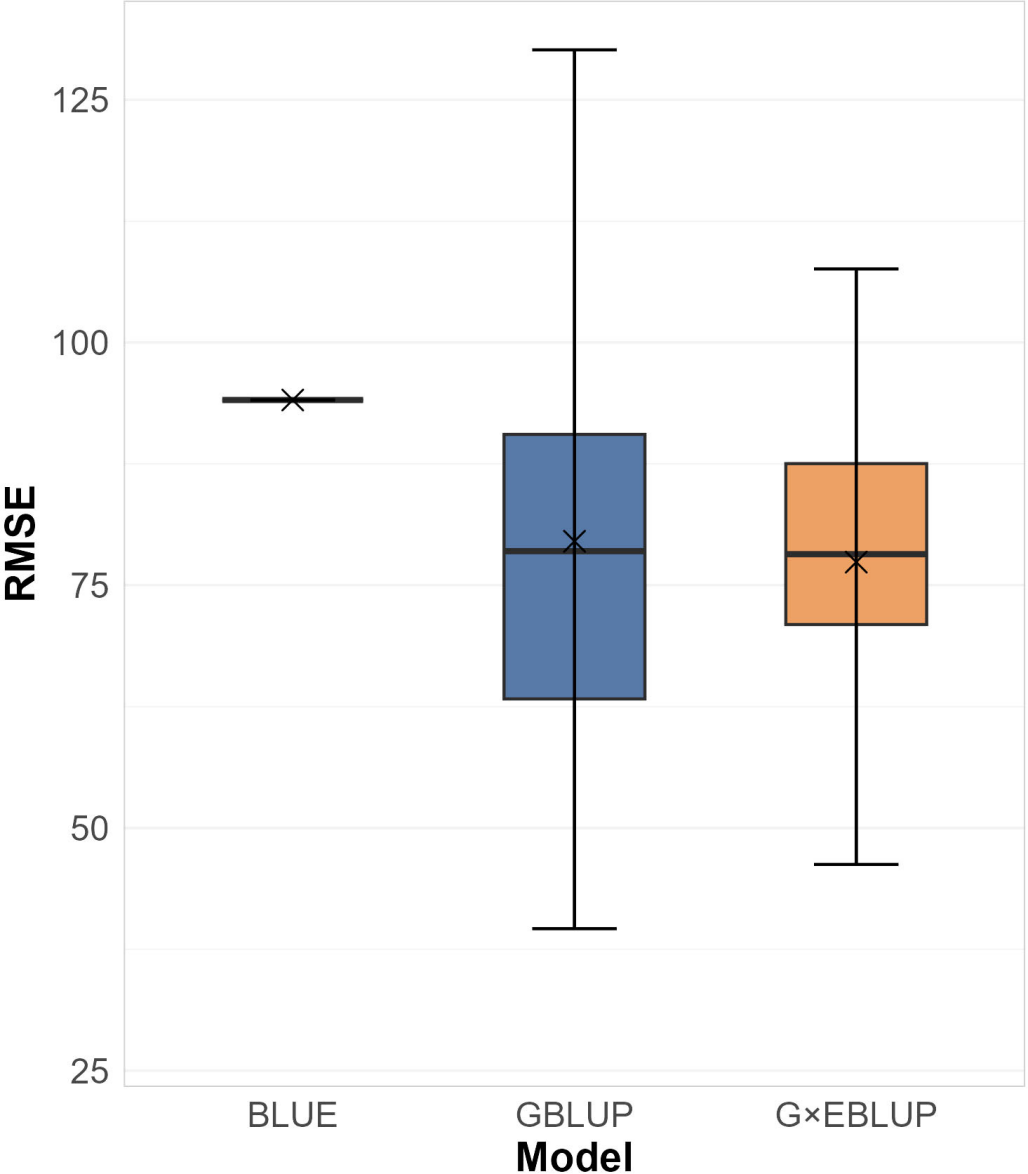
Box plots of Root Mean Square Error (RMSE) of the best identified BLUE, GBLUP, and G×EBLUP models by 72 leave-one-out cross-validation folds. Mean values are marked with ‘×’; median values are the middle lines.

Three sets of evaluation models were fitted and assessed, including G×EBLUP, GBLUP, and BLUE (**Table 2**). Comparing the best model identified in each set, the G×EBLUP framework achieved the highest CoD (0.925), marginally outperforming the GBLUP framework (CoD: 0.924) and BLUE framework (CoD: 0.888). The G×EBLUP model also had the lowest average RMSE of prediction (**Figure 2**), and its RMSEs were less variable across genotypes compared to the GBLUP model. Both the G×EBLUP and GBLUP models show lower mean and median RMSEs than the BLUE model.

**Table 2:** Comparison of statistical models for ryegrass dry matter yield across south-eastern Australian pasture environments.

Model	Best Model Components	logLik	AIC	BIC	MSE	CoD	PCC	Regions/Mega-environments	CCC
BLUE	y=Yield $X\beta =X_{BLUE}\beta_{BLUE}$ Zg is NA	-230523.69	462439.37	467756.93	8850.75	0.888	0.476	Tasmania	0.434
							0.498	South West Victoria	
							0.664	Gippsland	
							0.314	South Australia	
							0.443	Northern Victoria and Southern Riverina	
GBLUP	y=Yield $X\beta =X_{GP}\beta_{GP}$ $g\sim N(0,K_{0})$ and in $K_{0}$ : $\text{COR}({\text{λ}}_{Har})$ is order-three autoregressive variance structure	-225254.83	450713.66	451595.90	6048.22	0.924	0.521 ± 0.015	NA	0.489 ± 0.045
G×EBLUP	y=Yield $X\beta =X_{GP}\beta_{GP}$ $g\sim N(0,K_{1})$ and in $K_{1}$ : $\text{COR}({\text{λ}}_{Har})$ is order-three autoregressive variance structure, $\Gamma_{Tri}$ is order-two factor analytic loadings	-212617.24	425382.47	426016.04	5784.21	0.925	0.596 ± 0.014	AUM	0.542 ± 0.042
							0.614 ± 0.014	TAS	

Model comparisons include: (i) BLUE (Best Linear Unbiased Estimation) without incorporating genomic relationships and G×E (Genotype by Environment) interactions; (ii) GBLUP (Genomic Best Linear Unbiased Prediction) only incorporating additive genetic effects via genomic relationships; and (iii) GBLUP considering G×E interactions (G×EBLUP).

For a genomic prediction model using Equations 2 and 3, model performance is assessed using logLik, AIC, BIC, MSE, CoD, PCC, and CCC.

NA, Not Applicable.

The G×EBLUP framework presents the best-fitting model with the highest logLik (-212,617) and lowest AIC (425,382), BIC (426,016), and MSE (5784.21). Its optimal variance component combined an FA2 structure for G×E interactions and an order-three autoregressive structure for temporal correlations.

The best model identified in the GBLUP framework showed the second-best fit (logLik: -225,254, AIC: 450,713, BIC: 451,595, MSE: 6048.22), with order-three autoregressive structure as optimal temporal structure.

The best model from the BLUE framework showed the poorest fit (logLik: -230,523, AIC: 462,439, BIC: 467,756, MSE: 8850.75). The goodness-of-fit, as measured by CoD, was similar across frameworks.

The G×EBLUP framework showed superior prediction accuracy and precision with PCC ranging from 0.582-0.610 for AUM and 0.600-0.628 for TAS, and CCC ranging from 0.500-0.584. The GBLUP framework showed lower prediction accuracy and precision with PCC ranging from 0.506-0.536 and CCC from 0.444-0.534. The BLUE framework showed considerable precision variation across regions, with Gippsland achieving the highest precision (PCC: 0.664) and South Australia the lowest (PCC: 0.314).

### Dry matter yield prediction and estimation

The G×EBLUP model with the variance structures defined by $K_{1}$ in **Table 2** predicted seasonal DMY (DM kg/ha/season) for the 72 base ryegrass cultivars across 23 environments in Tasmania and the Australia mainland. The full prediction information is provided in **Supplementary Table S3**.

**Figure 3:**
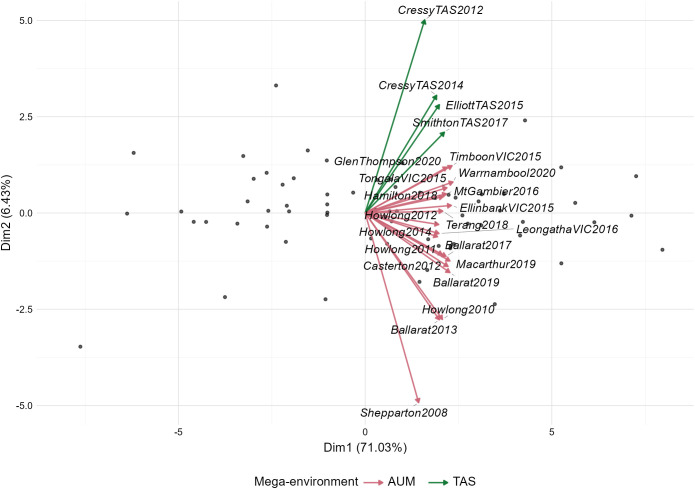
Biplot illustrating the Genotype × Environment interaction patterns across trial sites in Tasmania (TAS) and Australian mainland (AUM) mega-environments. Trials are represented as arrows, with green arrows indicating TAS environments and red arrows showing AUM environments. The relative angles between vectors indicate correlation strength between trials. Black dots represent individual genotype responses across environments.

The biplot (**Figure 3**) visualized the prediction, where the first two principal components explained 77.46% of the total variation in genotype responses across environments. The environments (arrows) formed two distinct clusters in the biplot. One group located in the upper quadrant consisted of TAS mega-environment (CressyTAS2012, CressyTAS2014, ElliottTAS2015, and SmithtonTAS2017). The other group AUM positioned in the mid and lower quadrants including Howlong, Ballarat, and Shepparton across years 2008-2020, with Shepparton2008 showing the greatest deviation from other environments.

The DMY performance differed across the two mega-environments (**Table 3**). In the AUM mega-environment, the highest annual DMY was achieved by BL011 (8263.5 ± 77.1 DM kg/ha/year), followed by Shogun (8226.6 ± 65.3) and Base (8187.2 ± 48.4). The lowest DMY were recorded for Victorian (6957.6 ± 47.8), Helix (6970.4 ± 73.6), and Endure (7072.3 ± 74.0). The range of annual DMY in AUM was 1305.9 DM kg/ha/year. In the TAS environment, Platinum achieved the highest annual DMY (7086.8 ± 138.7 DM kg/ha/year), followed by Shogun (7074.1 ± 139.1) and Avalon (7051.7 ± 91.3). The lowest performing cultivars were Endure (5330.5 ± 128.8), Meridian (5332.7 ± 144.0), and Helix (5623.1 ± 141.1). The TAS environment showed a larger range in annual DMY of 1756.3 DM kg/ha/year. Notably, Shogun maintained high performance in both mega-environments, while Helix and Endure consistently performed poorly.

**Table 3:** The seasonal and annual GBLUP (Genomic Best Linear Unbiased Prediction in DM kg/ha) of the top and bottom five base cultivars (corrected for endophyte effects) in the mega-environments (megaE) of AUM and TAS, with standard errors (SE); the number of the trials and harvests (Trial;Harvest) for each base cultivar in the mega-environment; and the least significant differences (LSD) within and across seasons at a 5% significance level.

megaE		Winter		Early Spring		Late Spring		Summer		Autumn		Annual	
	Base Cultivar	GBLUP(± SE)	Trial;Harvest	GBLUP(± SE)	Trial;Harvest	GBLUP(± SE)	Trial;Harvest	GBLUP(± SE)	Trial;Harvest	GBLUP(± SE)	Trial;Harvest	GBLUP(± SE)	Trial;Harvest
AUM	Top Five												
	BL011	1328.8 (± 41.9)	1;4	1844.9 (± 33.4)	1;6	2189.5 (± 28.5)	1;5	1661.2 (± 29.9)	1;4	1239.0 (± 36.9)	1;5	8263.5 (± 77.1)	1;24
	Shogun	1282.8 (± 35.3)	9;28	1969.8 (± 27.8)	9;40	2118.2 (± 24.0)	9;52	1695.7 (± 25.6)	9;52	1160.1 (± 31.8)	9;46	8226.6 (± 65.3)	9;218
	Base	1340.4 (± 26.0)	19;56	1793.9 (± 20.4)	19;80	2129.8 (± 18.4)	19;110	1664.6 (± 19.3)	19;106	1258.6 (± 23.2)	19;83	8187.2 (± 48.4)	19;435
	BL008	1281.7 (± 40.8)	2;8	1832.6 (± 32.4)	2;11	2151.7 (± 27.5)	2;11	1633.5 (± 28.8)	2;7	1226.3 (± 36.0)	2;11	8125.8 (± 74.8)	2;48
	Maxsyn	1296.7 (± 37.0)	4;13	1797.3 (± 29.2)	4;17	2133.5 (± 25.2)	4;26	1646.9 (± 26.9)	4;29	1222.7 (± 31.7)	4;14	8097.1 (± 67.7)	4;99
	Bottom Five												
	Kingsgate	1133.9 (± 39.7)	2;5	1642.4 (± 31.0)	2;5	2031.8 (± 27.0)	2;10	1452.8 (± 28.6)	2;11	1037.5 (± 35.5)	2;9	7298.3 (± 73.1)	2;40
	Meridian	1116.8 (± 41.8)	1;4	1674.0 (± 33.2)	1;5	1993.0 (± 28.4)	1;5	1464.1 (± 29.5)	1;1	1034.1 (± 36.4)	1;1	7282.1 (± 76.5)	1;16
	Endure	1066.7 (± 40.2)	3;9	1627.9 (± 32.2)	3;16	1942.6 (± 27.5)	3;18	1440.5 (± 28.5)	3;14	994.7 (± 35.4)	3;14	7072.3 (± 74.0)	3;71
	Helix	1066.3 (± 40.4)	3;12	1613.5 (± 32.1)	3;16	1919.5 (± 27.2)	3;16	1417.2 (± 28.1)	3;8	954.0 (± 35.2)	3;12	6970.4 (± 73.6)	3;64
	Victorian	958.1 (± 26.0)	20;68	1748.0 (± 20.7)	20;97	2107.9 (± 17.1)	20;131	1217.3 (± 18.9)	20;133	926.3 (± 23.1)	20;101	6957.6 (± 47.8)	20;530
	Summary												
	Mean	1208.4		1751		2082.8		1558.8		1124.6		7725.6	
	Range	382.3		356.3		270		478.4		332.3		1305.9	
	LSD	103.4		82.5		70.4		74.5		92.1		(within Seasons)	
		84.5										(across Seasons)	
TAS	Top Five												
	Platinum	1039.8 (± 69.5)	1;1	1432.0 (± 74.9)	1;3	2007.2 (± 47.6)	1;6	1466.0 (± 56.3)	1;5	1141.8 (± 58.0)	1;5	7086.8 (± 138.7)	1;20
	Shogun	976.2 (± 69.7)	1;1	1402.6 (± 75.1)	1;3	2096.3 (± 47.7)	1;6	1505.8 (± 56.4)	1;5	1093.2 (± 58.2)	1;5	7074.1 (± 139.1)	1;20
	Avalon	948.6 (± 51.8)	3;8	1398.0 (± 47.0)	3;7	2055.0 (± 30.0)	3;16	1536.1 (± 35.2)	3;13	1113.9 (± 36.2)	3;12	7051.7 (± 91.3)	3;56
	Base	1137.2 (± 51.6)	3;8	1238.1 (± 46.9)	3;7	1839.9 (± 29.9)	3;16	1646.8 (± 35.0)	3;13	1167.2 (± 36.1)	3;12	7029.2 (± 91.0)	3;56
	Kidman	976.4 (± 78.1)	2;7	1398.6 (± 59.5)	2;4	1988.9 (± 39.3)	2;10	1526.8 (± 44.9)	2;8	1111.3 (± 45.9)	2;7	7001.9 (± 123.7)	2;36
	Bottom Five												
	Ohau	755.1 (± 85.6)	1;3	1269.6 (± 71.7)	1;2	1828.3 (± 47.0)	1;5	1379.0 (± 52.3)	1;3	945.4 (± 54.3)	1;3	6177.4 (± 142.7)	1;16
	Boomer	666.2 (± 86.3)	1;3	1333.0 (± 72.4)	1;2	1783.0 (± 47.4)	1;5	1269.0 (± 52.8)	1;3	873.4 (± 54.8)	1;3	5924.7 (± 143.9)	1;16
	Helix	686.9 (± 84.7)	1;3	1195.2 (± 70.9)	1;2	1724.4 (± 46.4)	1;5	1256.4 (± 51.6)	1;3	760.2 (± 53.7)	1;3	5623.1 (± 141.1)	1;16
	Meridian	591.9 (± 86.3)	1;3	1064.4 (± 72.4)	1;2	1812.4 (± 47.4)	1;5	1139.4 (± 52.8)	1;3	724.6 (± 54.8)	1;3	5332.7 (± 144.0)	1;16
	Endure	567.7 (± 81.1)	2;7	1121.8 (± 62.0)	2;4	1700.6 (± 41.0)	2;10	1115.2 (± 46.9)	2;8	825.1 (± 47.8)	2;7	5330.5 (± 128.8)	2;36
	Summary												
	Mean	901.5		1352.2		1896.9		1437.4		1022.3		6610.4	
	Range	569.4		548.7		413.7		531.5		442.6		1756.3	
	LSD	226.6		205.5		132.9		154.5		158.7		(within Seasons)	
		175.4										(across Seasons)	

Seasonal variations were also observed in both mega-environments. In AUM, the seasonal means followed the order of Late Spring (2082.8 DM kg/ha/season) > Early Spring (1751.0) > Summer (1558.8) > Winter (1208.4) > Autumn (1124.6). The differences between all seasons were significant based on the LSD (84.5 DM kg/ha across seasons). In Late Spring, BL011 achieved the highest DMY (2189.5) while Helix had the lowest (1919.5). In the lowest-yielding season (Autumn), Base performed best (1258.6) while Victorian yielded lowest (926.3). In TAS, the seasonal means also showed significant differences (LSD = 175.4 DM kg/ha across seasons) with the order being Late Spring (1896.9) > Summer (1437.4) > Early Spring (1352.2) > Autumn (1022.3) > Winter (901.5). WintasII achieved the highest Late Spring DMY (2114.3) while Endure had the lowest (1700.6). In Winter, the lowest-yielding season in TAS, Base performed best (1137.2) while Endure again showed the lowest yield (567.7) (**Supplementary Table S3**).

### Genomic estimated breeding values and heritability

Genomic prediction models revealed moderate narrow-sense heritability, with *h²* = 0.31 for the model without G×E and *h²* = 0.39 for the model including G×E interactions. Broad-sense heritability was high in both environments (*H²* = 0.73 in AUM and *H²* = 0.74 in TAS), with genetic variances of the environments accounting for 34% and 35% of total variance, respectively. The GEBVs of base cultivars ranged from -813.59 to 524.44 DM kg/ha/season (**Supplementary Table S4**). The top five cultivars based on GEBVs were BL011 (524.44), Shogun (506.07), Base (441.11), BL008 (406.99), and Maxsyn (359.69).

**Figure 4:**
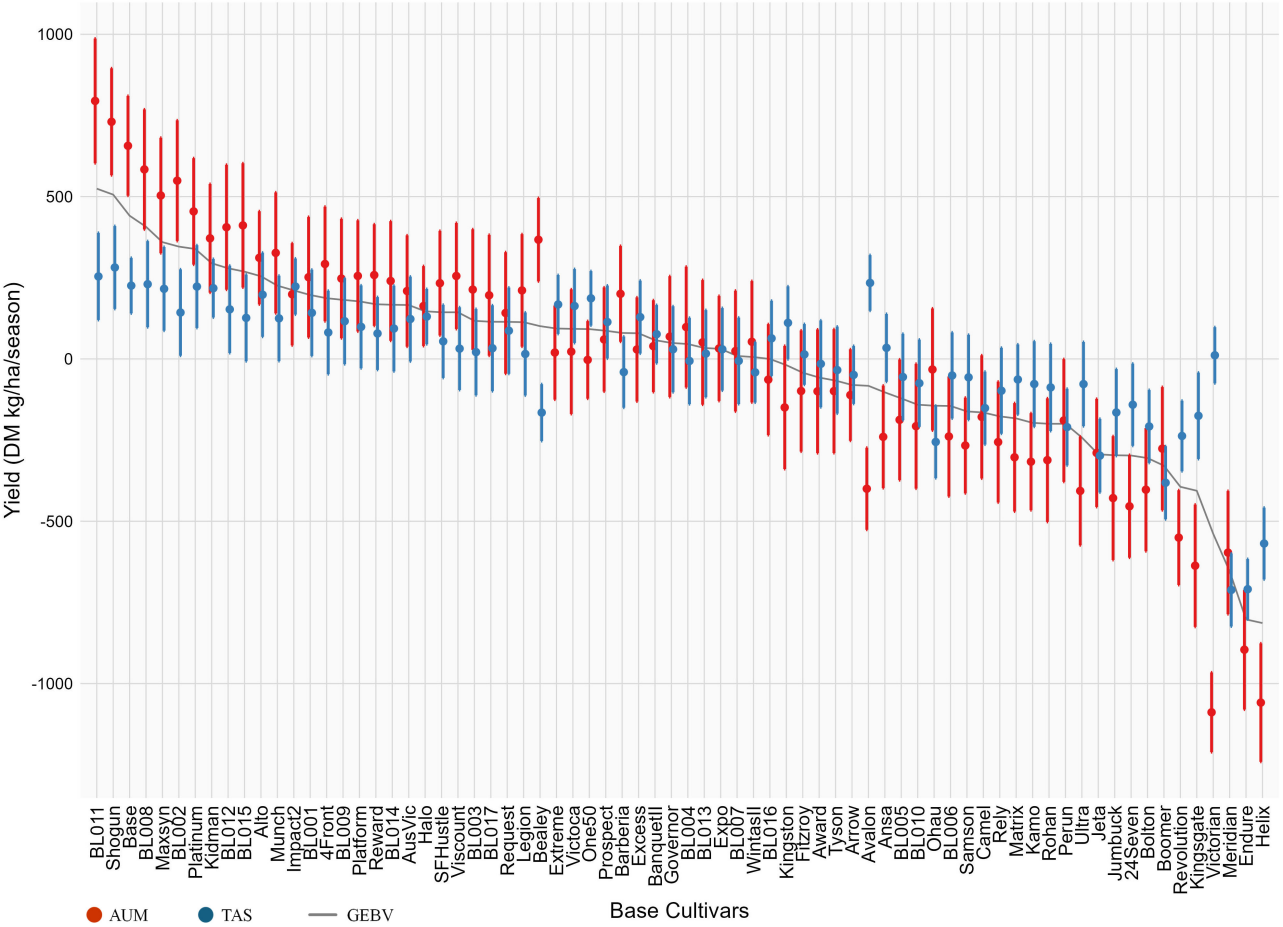
The Genomic Estimated Breeding Values (GEBV: grey curve) and environment-driven genetic responses of base cultivars in two mega-environments (AUM: red, TAS: blue). The Error bars represent standard errors. Base cultivars are ordered by their GEBVs in descending order.

Environment-driven genetic responses varied between mega-environments. In AUM, DMY responses ranged from -1088.48 (Victorian) to 794.75 (BL011) DM kg/ha/season. In TAS, the range was from -711.66 (Meridian) to 281.70 (Shogun) DM kg/ha/season. Most base cultivars maintained stable responses across environments. However, several base cultivars showed substantial re-ranking between mega-environments (**Figure 4** and **Supplementary Table S4**). For example, Avalon ranked 62nd for AUM response but 3rd for TAS response, while Bealey ranked 11th in AUM but 62nd in TAS.

## Discussion

### Spatial analysis and phenotyping

Accurate phenotyping is crucial for developing reliable GP models. This study utilized MHMS trials of perennial ryegrass across diverse environmental conditions to phenotype the DMY of the 72 ryegrass genotypes. However, within each trial, measuring DMY is challenging due to local spatial variation, highlighting the necessity of spatial analysis within trials in our study.

Local spatial variation within trials involves soil heterogeneity, local moisture gradients, fertility differences, or management practices (Gilmour et al., 1997; Piepho et al., 2008) and can introduce biases in genetic responses in GP models, reducing model precision. Traditional experimental designs, like randomized complete block designs, often fail to fully address the biases, especially in large-scale multiple trials (Gilmour et al., 1997; Hoefler et al., 2020; Piepho et al., 2008; Smith et al., 2005). Advanced spatial analysis methods using mixed two-dimensional covariance structures in this study mitigated these spatial confounding effects and improve GP precision.

The models, where the spatial effects were fitted as both fixed and random effects, were used to address field heterogeneities without assuming linear trends along rows and columns. Cultivar effects were treated as fixed to retain raw field responses at the individual data point level (e.g., each replicate per harvest). This approach ensures results comparable to single-stage analysis (Holland and Piepho, 2024) without misusing BLUP multiple times and corrects phenotypes for spatial biases, making them suitable for genomic modelling. Besides, comparisons of spatial modelling approaches indicated that autoregressive structures outperformed antedependence structures in computational efficiency.

To better account for field heterogeneities, the implementation of unmanned aerial vehicles with multispectral sensors presents an opportunity for the quantification of additional agronomical traits beyond DMY (Gebremedhin et al., 2020; Pranga et al., 2021; Tanaka et al., 2024; Wang et al., 2019). Plus, the development of non-destructive phenotyping methodologies would facilitate high-throughput data acquisition without impacting the integrity of the cultivars under evaluation (Ludovisi et al., 2017; Rahaman et al., 2015). Such technological advancements would be particularly effective for the temporal characterisation of DMY fluctuations throughout the growing season (Nguyen et al., 2022; Wang et al., 2019).

### Genomic relationship analysis

Sufficient marker density of genotyping is often necessary to cover the short linkage disequilibrium (LD) present in ryegrass chromosomes and maintain high predictive abilities (Arojju et al., 2020b; Barre et al., 2022; Guo et al., 2018; Lin et al., 2016). This study utilised a dataset from a previous investigation (Zhu et al., 2025), which employed a target sequencing approach and identified ~86k high-density SNPs to explore the genomic relationships among the 72 ryegrass genotypes. Notably, pool sequencing methodology was employed (Zhu et al., 2025), wherein each cultivar was represented by at least 50 individual plants, with several genotypes comprising multiple cultivars (sharing genetic backgrounds but differing in endophyte combinations). This approach quantified genetic variance within each genotype using allele frequencies rather than discrete encoding (such as 0/1/2), thereby effectively representing population-level variation across more than 40k plants. This representation was particularly valuable for depicting heterozygosity within populations and the underlying genetic complexity of outcrossing, polyploid species like perennial ryegrass (Guo et al., 2018; Zhu et al., 2025).

The construction of the GRM followed the methods by Yang et al. (2010) and was adapted for allele frequency encoding (Equation 1). This method enables the modelling of additive genetic relationships from genome-wide SNP data without assuming Hardy-Weinberg equilibrium, making it well-suited to outbred species like perennial ryegrass, which exhibit high heterozygosity and complex breeding histories (Arojju et al., 2018; Barre et al., 2022; Fè et al., 2015; Hayes et al., 2013; Yang et al., 2010). The GRM captured both historical recombination and recent breeding divergence among cultivars, implicitly accounting for population structure without requiring explicit stratification correction. This is especially relevant given the genetic diversity of the ryegrass base cultivars in this study, which originated from different breeding programs. The suitability of the GRM was demonstrated by the negative inbreeding coefficients (F < 0) observed in the majority of germplasms, reflecting historical crosses between genetically distinct populations. In contrast, the cultivar Barberia showed a positive inbreeding coefficient (F > 0), indicating reduced genetic diversity likely due to strong selection. These patterns support the effective integration of genomic relationships into the DMY prediction framework implemented in this study.

However, it is important to recognize that high marker densities are not always a cost-effective option for perennial ryegrass evaluation and selection programs. Studies have demonstrated that prediction accuracies plateau through LD-pruning or targeted SNP selection (Arojju et al., 2020b; Song and Hu, 2022). Furthermore, optimized low-density SNP arrays coupled with well-designed imputation algorithms (e.g. Wu et al., 2016) could halve genotyping costs with minor losses in predictive ability. Therefore, a balanced approach of tailoring marker density to genetic architectures and genotyping cost could ensure efficient GP without unnecessary expenditure.

### Integrating genomic relationships into DMY estimation

This study first explored and integrated genomic relationships along with large-scale MHMS field trials to improve the DMY estimation in a perennial ryegrass regional evaluation system. By leveraging genomic relationships, GP enabled estimations of additive genetic variances and the prediction of DMY for untested cultivars, even when they were not present in any trials or environments. Such feasibility was validated by LOOCV in independently simulating scenarios where DMY of a certain cultivar is predicted via a genomic relationship without actual measurements. This demonstrates the potential to predict DMY using genotyping data alone, potentially reducing the need for costly and time-consuming field trials.

The GBLUP model in this study incorporated genomic information through the GRM, which reveals pseudo-pedigree relationships among the genotypes. Usage of GRM kernel was also a consideration of both computational efficiency and proven predictive accuracies in the GP applications by other studies (Arojju et al., 2020b; Cericola et al., 2018; Faville et al., 2021, 2018; Fè et al., 2016, 2015; Jahufer et al., 2021; Konkolewska et al., 2023; Lin et al., 2016).

Incorporating genomic data improved predictive accuracy and precision, as demonstrated by increased CCCs from the baseline BLUE (0.434) to the GBLUP (0.489), resulting in a 12.7% improvement. This was further evidenced by a 56.9% reduction in the average standard error of 46.88 DM kg/ha/season in the current study from the average standard error of 108.75 DM kg/ha/season reported by Zhu et al. (2023), where BLUP modelled G×E interactions but did not incorporate genomic data.

This study also highlighted the need to separate endophyte symbiotic impacts on host plant genetic responses when evaluating perennial ryegrass performance. This separation is critical because endophytes significantly impact the genetic responses of the ryegrasses. Additionally, endophytes are typically confined to specific cultivars due to commercial agreements between endophyte owners and ryegrass breeding companies, creating an imbalanced dataset where not all endophyte-ryegrass combinations can be tested (Zhu et al., 2025).

### Environmental enhanced ryegrass evaluation system for DMY performance

In Australia, farmers face significant challenges in selecting from over 60 commercially available perennial ryegrass cultivars (Leddin et al., 2018). Wherein, current industry standard, which presents an aggregate BLUE across trials, incorporates seasonal performance weighted by the relative economic value across regions. This approach assumes consistent genotype differences within and between regions. However, our analysis reveals substantial variation in estimation precision across regions (PCC ranging from 0.314 in South Australia to 0.664 in Gippsland) when using BLUE. This inconsistency in precision strongly indicates the presence of underlying biological G×E interactions that the current economic-based evaluation system inadequately captured.

By explicitly modelling biological G×E interactions through the G×EBLUP, we achieved more stable predictive ability across the identified mega-environments (PCC: 0.596 ± 0.014 for AUM and 0.614 ± 0.014 for TAS). Besides, predicting DMY based on specific mega-environments improved the evaluation reliability, as evidenced by an overall 24.9% increase in CCC from the BLUE (0.434) to the G×EBLUP (0.542), demonstrating marked improvement over current industry approaches. In addition, the BLUP framework maintains practical utility in effectively accounting for complex genetic variance components with unbalanced datasets (Robinson, 1991), also facilitating accurate predictions.

The shift from GBLUP to G×EBLUP resulted in an additional 10.8% increase in predictive abilities (CCC from 0.489 to 0.542) and an improved narrow-sense heritability from 0.31 to 0.39. This is because perennial ryegrass DMY is a complex quantitative trait influenced by multiple genetic and environmental factors; the G×EBLUP model, which extended GBLUP by modelling G×E interactions through the $K_{1}$ structure better modelled such complexity. The improvement was also comparable to other studies which reported low to moderate prediction accuracies (Bornhofen et al., 2022; Faville et al., 2018; Grinberg et al., 2016; Jahufer et al., 2021; Konkolewska et al., 2023; Pembleton et al., 2018), where even though G×E interactions were not fully explored due to their limited multi-environmental phenotyping datasets.

Furthermore, environmental evaluation revealed significant variations in DMY across the two identified mega-environments, AUM and TAS, which align geographically with mainland Australia and Tasmania, respectively. Seasonal fluctuations were evident, with Late Spring producing the highest DMY in both mega-environments, while Winter and Autumn displayed lowest DMY in both mega-environments. The mega-environment TAS exhibited greater uncertainty in DMY prediction, as indicated by larger LSDs, emphasizing the need for a larger dataset than the current compared to AUM. These findings highlight the necessity of representative field trials encompassing both geographical and temporal dimensions in improving the accuracy of perennial ryegrass DMY prediction.

### Regional adaptation patterns of perennial ryegrass

Breeding value estimation, based on GEBVs, provides insights into the genetic potential that passes from breeding lines to their progeny. This approach offers great advantages over traditional breeding methods, which primarily rely on phenotypic recurrent selection, usually require more than a decade per cycle, and struggle to accurately evaluate potentials across diverse environments (Barre et al., 2022; Hayes et al., 2013; Lin et al., 2016).

Environment-driven genetic responses explored in this study revealed the variability in genetic potentials under different environmental conditions, largely attributed to non-additive genetic effects related to G×E interactions, such as dominance and epistasis effects (Duenk et al., 2020; Su et al., 2012; Varona et al., 2018). Our analysis identified distinct patterns across regions, with AUM showing wider variations in environmental responses while TAS displayed more stable genetic responses. These regional differences were further exemplified by genotype-specific adaptation patterns where Avalon demonstrated strong adaptation in TAS, Bealey excelled in AUM, and Shogun exhibited broad adaptability across both mega-environments.

The variability in the environment responses can stem from multiple sources of environmental variation, including differences in climate characteristics, soil physical and chemical properties, and root development patterns (Chapman et al., 2017; Faville et al., 2018; Konkolewska et al., 2023; Wedderburn et al., 2010; Zhu et al., 2023). Temporal variation, encompassing both seasonal and inter-annual fluctuations, further complicates predictions (Colas et al., 2022; Gilliland and Hennessy, 2021; Giri et al., 2019; Robins and Alan Lovatt, 2016). Recent environmental profiling analysis by Zhu et al. (2023) identified key environmental drivers of G×E interactions in perennial ryegrass, revealing that soil properties, temperature, and evaporation rate were primary factors differentiating environmental clusters. These analyses demonstrated that both soil-related characteristics and weather-related factors contributed to mega-environment differentiation that could be leveraged to enhance future modelling approaches and prediction accuracy.

These findings comprehensively demonstrated both the importance of integrating genomic relationships and accounting for G×E interactions when better estimating perennial ryegrass DMY in regional evaluation systems. They also emphasized the need for environment-specific implementation strategies that operate independently of economic interests to meet regional demands for reliable evaluation of ryegrass productivity and genetic gains.

### Implementation strategies

The influence of G×E interactions necessitate environment-specific strategies. In Tasmania, water-responsive or cold-climate varieties such as Platinum, Shogun, and Avalon are recommended for their superior performance. On the mainland, stable high-yielding cultivars like Shogun and Base are prioritized to accommodate diverse environmental conditions. These targeted recommendations align cultivar traits with regional needs to maximize productivity.

Seasonal variation is another critical dimension to consider. Late Spring usually offers peak DMY, while Winter and Autumn conditions limit performance. Management strategies may include maximizing annual harvests and implementing adaptive practices for production systems, such as altering calving dates to better match pasture supply and animal demand. These tailored approaches could optimize sustained productivity year-round.

When breeding new elite cultivars, a dual strategy is suggested to balance genetic gain and diversity. Initial selection should leverage GEBVs to capitalise on those additive genetic potential with moderate heritability. Crossbreeding designs could then include close-family crosses to maximize genetic gain through hybrid vigour or far-family crosses to maintain genetic diversity, to ensure short-term performance improvements and long-term sustainability.

While GP offers a powerful approach to evaluate genetic gain, it should be viewed as an integrative component within established perennial ryegrass evaluation and breeding frameworks, including F2 Family (Bornhofen et al., 2022; Cericola et al., 2018; Fè et al., 2016, 2015), Half-Sib Family (Arojju et al., 2020a; Faville et al., 2018; Jahufer et al., 2021), and Synthetic Population approaches (Faville et al., 2016; Hayes et al., 2013; Malmberg et al., 2023; Pembleton et al., 2018). Effective implementation of GP relies on well-designed breeding programs with regionally representative trials, advanced phenotypic technologies, and strategic integration of genomic information across these frameworks. The absence of a centralized and coordinated pasture evaluation system in Australia presents structural challenges that GP alone cannot resolve. Therefore, realising the full potential of GP may require alignment with a more structured and collaborative evaluation framework that considers other agronomically important traits beyond DMY, such as nutritive traits (Leddin et al., 2022) and metabolizable energy (Lewis et al., 2024).

In future research, GP models could be further enhanced by incorporating additional data sources to address non-additive genetic variances or those unexplained variances stemming from environmental and management factors. These may include climate variables, soil-genotype interactions, and practices such as irrigation, fertilization, and grazing management (Fiorelli et al., 2001; Peters et al., 2022). By integrating these aspects, models may achieve greater accuracy to better reflect real-world complexities. For instance, incorporating plant growth models, such as APSIM (Agricultural Production Systems Simulator), could enhance predictions by simulating genotype responses to environmental factors dynamically (Hammer et al., 2023).

In conclusion, productivity estimation and genomic prediction require continuous refinement as new data becomes available, and their reliability must be validated through large-scale trials before implementation. This validation will not only assess predictive accuracy under real-world conditions but also evaluate the economic feasibility of future breeding. Through the systematic evaluation and validation process discussed in this study, breeding programs can adapt to changing agricultural conditions and effectively meet regional demands, ultimately supporting sustainable agricultural practices across diverse pasture environments.

## Data Availability

Publicly available datasets were analysed in this study. This data can be found here: Ryegrass_Genotype_Allele_Frequency_Dataset. The University of Melbourne research repository. https://doi.org/10.26188/26392210.v1.

## Supplementary material

Supplementary Material 1 The report of tested spatial models of the six spatial frameworks across the 23 trials.

Supplementary Table 2 The details about all tested spatial models of the six spatial frameworks and their performances across the 23 trials.

Supplementary Table 3 Seasonal and annual predictions for all base cultivars across the two identified mega-environments, AUM and TAS, based on the G×EBLUP model.

Supplementary Table 4 The GEBVs and environment-driven genetic responses.
