# Supplementary material for: Estimation of ryegrass (Lolium) dry matter yield using genomic prediction considering genotype by environment interaction across south-eastern Australia
Source: Front Plant Sci. 2025 Jun 9;16:1579376. doi: 10.3389/fpls.2025.1579376 (PMC12183291; doi:10.3389/fpls.2025.1579376)
Supplement: Supplementary Material 1 — The report of tested spatial models of the six spatial frameworks across the 23 trials. [file DataSheet1.pdf]

Supplementary Material

Supplementary Material 1: The report of tested spatial models of the six spatial frameworks across the 23 trials.

Statistical Methods

The models included Base (no spatial effects were accounted for), Spatial Fixed (the spatial effects were fitted as fixed effects), Spatial Fixed Linear (the spatial effects were assumed to have a fixed linear trend), Spatial Random (the spatial effects were fitted as random effects following a distribution as Table S1), Spatial Mixed (the spatial effects were fitted as both fixed and random effects following a distribution as Table S1), and Spatial Mixed Linear (the spatial effects were fitted as both fixed and random effects and the fixed spatial effects were assumed to having a fixed linear trend). In general, all frameworks maintain a structure of

$$\boldsymbol{y} = \boldsymbol{X\beta} + \boldsymbol{Zu} + \boldsymbol{\varepsilon}$$

where, $\boldsymbol{y}$ is the vector of observations; $\boldsymbol{X}$ and $\boldsymbol{Z}$ are design matrices for fixed ($\boldsymbol{\beta}$) and random effects ($\boldsymbol{u} \sim \mathcal{N}(0, \boldsymbol{U})$), respectively; $\boldsymbol{\varepsilon}$ is the vector of residual errors, $\boldsymbol{\varepsilon} \sim \mathcal{N}\big(0, \mathrm{Var}(\boldsymbol{\varepsilon})\big)$.

Specifically, each framework was given as

Supplementary Table 1. The six frameworks of spatial models per *Trial*.

Framework	Xβ	Zu
Base	$(\mu, \boldsymbol{Cul}\vert\boldsymbol{Har})\big(1, \boldsymbol{\beta}_{Cul\vert Har}\big)'$	
Spatial Fixed	$(\mu, \boldsymbol{Cul}\vert\boldsymbol{Har}, \boldsymbol{Row}\vert\boldsymbol{Har}, \boldsymbol{Col}\vert\boldsymbol{Har})\big(1, \boldsymbol{\beta}_{Cul\vert Har}, \boldsymbol{\beta}_{Row\vert Har}, \boldsymbol{\beta}_{Col\vert Har}\big)'$	NA
Spatial Fixed Linear	$(\mu, \boldsymbol{Cul}\vert\boldsymbol{Har}, \boldsymbol{Row}\vert\boldsymbol{Har}, \boldsymbol{Col}\vert\boldsymbol{Har})\big(1, \boldsymbol{\beta}_{Cul\vert Har}, \boldsymbol{\beta}_{lin(Row)\vert Har}, \boldsymbol{\beta}_{lin(Col)\vert Har}\big)'$	
Spatial Random	$(\mu, \boldsymbol{Cul}\vert\boldsymbol{Har})\big(1, \boldsymbol{\beta}_{Cul\vert Har}\big)'$	
Spatial Mixed	$(\mu, \boldsymbol{Cul}\vert\boldsymbol{Har}, \boldsymbol{Row}\vert\boldsymbol{Har}, \boldsymbol{Col}\vert\boldsymbol{Har})\big(1, \boldsymbol{\beta}_{Cul\vert Har}, \boldsymbol{\beta}_{Row\vert Har}, \boldsymbol{\beta}_{Col\vert Har}\big)'$	$\boldsymbol{U} = \left(\boldsymbol{\Sigma}_{Har}^{\mathrm{COR}(\lambda_{Har})}\right) \otimes \left(\boldsymbol{\Sigma}_{Row}^{\mathrm{COR}(\lambda_{Row})}\right) \otimes \left(\boldsymbol{\Sigma}_{Col}^{\mathrm{COR}(\lambda_{Col})}\right)$
Spatial Mixed Linear	$(\mu, \boldsymbol{Cul}\vert\boldsymbol{Har}, \boldsymbol{Row}\vert\boldsymbol{Har}, \boldsymbol{Col}\vert\boldsymbol{Har})\big(1, \boldsymbol{\beta}_{Cul\vert Har}, \boldsymbol{\beta}_{lin(Row)\vert Har}, \boldsymbol{\beta}_{lin(Col)\vert Har}\big)'$	

Where, μ is the *Intercept;* $\boldsymbol{Cul}|\boldsymbol{Har}$, $\boldsymbol{Row}|\boldsymbol{Har}$, *and* $\boldsymbol{Col}|\boldsymbol{Har}$ are design matrices for the fixed effect of *Cultivar* within *Harvest* ($\boldsymbol{\beta}_{Cul|Har}$), *Row* within *Harvest* ($\boldsymbol{\beta}_{Row|Har}$), and *Column* within *Harvest* ($\boldsymbol{\beta}_{Col|Har}$), respectively, or the linear combination of *Row* effects within *Harvest* ($\boldsymbol{\beta}_{lin(Row)|Har}$), and the linear combination *Column* effects within *Harvest* ($\boldsymbol{\beta}_{lin(Col)|Har}$), respectively; $\boldsymbol{\Sigma}_{Har}^{\mathrm{COR}(\lambda_{Har})}$, $\boldsymbol{\Sigma}_{Row}^{\mathrm{COR}(\lambda_{Row})}$, and $\boldsymbol{\Sigma}_{Col}^{\mathrm{COR}(\lambda_{Col})}$ are the variance-covariance matrices for *Harvest*, *Row*, and *Column*, respectively, with order-λ_{Har},

order-λ_{Row}, and order-λ_{Col} autoregressive or ante-dependence structures, $\lambda_{Har}, \lambda_{Row}$, or $\lambda_{Col} \in \{1,2,3\}$. $\boldsymbol{M}'$ means the transpose of $\boldsymbol{M}$. $\otimes$ denotes the Kronecker product.

Variance components including order-one, two, and three autoregressive covariance structures as well as order-one, two, and three ante-dependence covariance structures for *Harvest*, *Row*, and *Column* were tested. All models were assessed based on log-likelihood (logLik), Akaike Information Criterion (AIC), Bayesian Information Criterion (BIC), and Mean Absolute Error (MAE), to identify the most appropriate model for each trial.

Results

The performance of six spatial model frameworks was comprehensively evaluated across the 23 trials, with MAE assessment visualized in Fig. S1. The mixed-spatial frameworks (Spatial Mixed in mint green and Spatial Mixed Linear in yellow) demonstrated generally superior performance compared to the fixed- spatial frameworks (Spatial Fixed in navy blue and Spatial Fixed Linear in teal). Models that accounted for spatial effects without assuming linear trends along rows and columns outperformed those that incorporated linear trend assumptions, as evidenced by the comparison between Spatial Fixed versus Spatial Fixed Linear, and Spatial Mixed versus Spatial Mixed Linear frameworks. Notably, all spatial model frameworks exhibited better performance than the Base model (indicated by purple) that does not account for spatial variation. Based on these comparative analyses, the Spatial Mixed framework emerged as the optimal approach, from which the model generally with the largest logLik as well as the lowest AIC, BIC, and MAE across *Harvest*, *Row*, and *Column*, was selected to account for spatial effects consistently across the 23 trials (Supplementary TableS2).

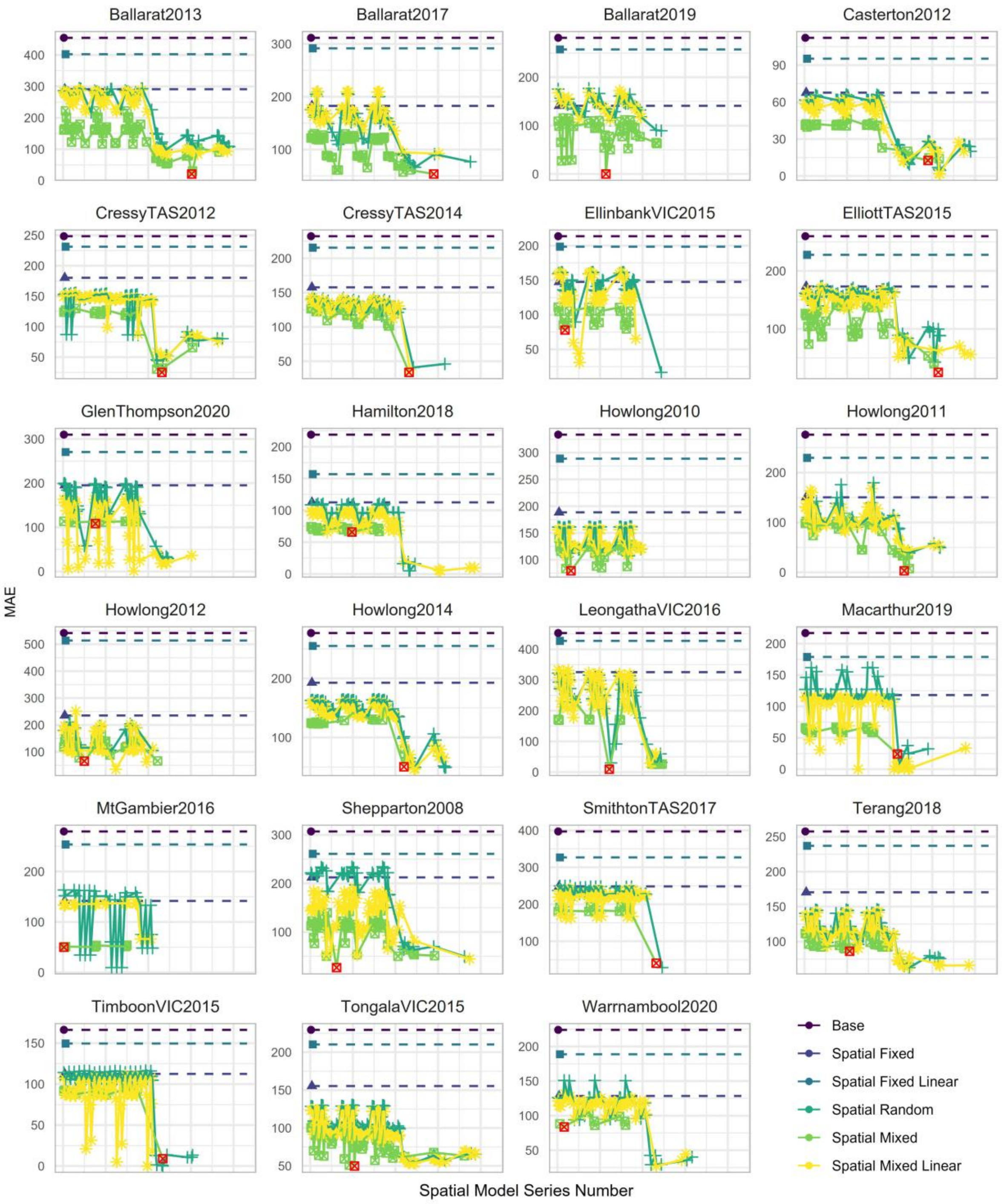

Supplementary Figure 1. Spatial Model performances by mean absolute error (MAE) for the 23 trials. Each facet represents a single trial. The model frameworks include the Base model, Spatial Fixed, Spatial Fixed Linear, Spatial Random, Spatial Mixed, and Spatial Mixed Linear models. The horizontal dashed lines represent single-point models' MAE values across the series. The best model identified for obtaining unbiased estimates of the spatial effects is marked by a red point for each trial. The x-axis is the spatial model series number, and the y-axis shows the MAE values.
